# A Case of Neuralgia

**Published:** 1850-07

**Authors:** A. S. Dwyer


For the Dental Register.

### A CASE OF NEURALGIA.

BY A. S. DWYER, D. D. S.

*Dr. Taylor,*

In compliance with your request I give you a history of Mrs. S.'s case, the lady from whom I extracted the teeth which I presented to you. Mrs. S. had been for a number of years afflicted with neuralgia of the head and face, partial paralysis of both her hands and arms; so much so that she could not raise her hands to her mouth, though she only lost the use of one arm and hand at a time. She had also been afflicted with leuchorrea, prolapsus uteri and uterine neuralgia, which had emaciated her so much that when I was called upon to examine her teeth, she had not been able to leave her room for a length of time; indeed, serious fears were entertained by many, that she would not recover. Her teeth appeared to be all diseased, though not all decayed; her gums were swollen, and presented a livid appearance, and were absorbed away from around the necks of the teeth, giving their edges a thick appearance; from the gums was a discharge of an exceedingly offensive character. She had been treated by a number of physicians, with all the various remedies their skill could suggest, but apparently in vain. Dr. I. L. Wilson, upon whom I called to be present, and assist, if necessary, (as I intended to exhibit to her chloroform, as she was very sensitive,) informed me that prior to that time one year, he had extracted one sup. molar which was much exostosed, and that a day or so previously, he had extracted a tooth which was much exostosed, and took much force to remove it—that after he had removed it, she became very much exhaused, approaching very near to syncope, in which state she remained for some time, and that suddenly she sprang up in the bed, convulsively clutching her hands in her hair; alternately shrieking and sobbing for a time, and at length piteously implored them to relieve her, that her head was in a vise—was surrounded with a cord, or that a heavy weight was resting upon her head, and that she became so frantic with pain, it was with difficulty he could control her, and that during these paroxysms she suffered severely from uterine neuralgia, and that her leuchorrea was much aggravated, and the family assured me that she had frequently suffered from such paroxysms every day for a length of time. After exhibiting to her the chloroform, so as to produce insensibility to the pain arising from extracting, on the 18th of May, 1849, I removed two sup. bicuspids, the ones so badly exostosed * and broke off an inferior bicuspid even with the alveolus. Again, on 3d of the next month, I removed four teeth, and on the sixth removed the sup. dens sapientiæ; it had never made its appearance, though the lady is between the age of 55 and 60. The teeth in front of it having been removed, gave the appearance of exostosis of the maxilla; when her jaws

* These teeth certainly exhibit as fine specimens of this disease as we have ever seen.

The entire case above described by our friend, Dr. Dwyer, forcibly illustrates the effect of dental irritation on the general system, and shows, also, the importance of a more general attention to the dental organs by our medical practitioners. We have no doubt but that all the teeth still remaining in the mouth are effected with the same disease, and that the general health will not be perfectly restored until they are removed.—J. T., Cin. Ed.

were closed, this portion came in contact with the under jaw. After giving her chloroform, I made a crucial incision, dissected up the flaps, and then took hold of what I thought might be a tooth, but only succeeded in bringing away a piece of the crown of a tooth; now knowing it to be a tooth, I took hold of it with a pair of sup. dens sapientiæ forceps, with which I removed it.

There are some five or six teeth in front above, yet remaining, which I intended to have removed before this time, but her health has improved so much that she has regained the use of her hands and arms, and is able to again resume her former occupation, (being a milliner,) that I have not been able as yet to induce her to have the remaining teeth extracted, though she says at times they cause her pain, and she will have them removed.

